# The impact of growth hormone on proteomic profiles: a review of mouse and adult human studies

**DOI:** 10.1186/s12014-017-9160-2

**Published:** 2017-06-29

**Authors:** Silvana Duran-Ortiz, Alison L. Brittain, John J. Kopchick

**Affiliations:** 1 Edison Biotechnology Institute, Ohio University, Athens, OH USA; 2 Department of Biological Sciences, College of Arts and Sciences, Ohio University, Athens, OH USA; 3 Molecular and Cellular Biology Program, Ohio University, Athens, OH USA; 4 Department of Biomedical Sciences, Heritage College of Osteopathic Medicine, Ohio University, Athens, OH 45701 USA

**Keywords:** Growth hormone, Human proteomics, Mouse proteomics, Aging, GHR^−/−^ mice, bGH mice, Growth hormone deficiency, Acromegaly, Growth hormone doping

## Abstract

Growth hormone (GH) is a protein that is known to stimulate postnatal growth, counter regulate insulin’s action and induce expression of insulin-like growth factor-1. GH exerts anabolic or catabolic effects depending upon on the targeted tissue. For instance, GH increases skeletal muscle and decreases adipose tissue mass. Our laboratory has spent the past two decades studying these effects, including the effects of GH excess and depletion, on the proteome of several mouse and human tissues. This review first discusses proteomic techniques that are commonly used for these types of studies. We then examine the proteomic differences found in mice with excess circulating GH (bGH mice) or mice with disruption of the GH receptor gene (GHR^−/−^). We also describe the effects of increased and decreased GH action on the proteome of adult patients with either acromegaly, GH deficiency or patients after short-term GH treatment. Finally, we explain how these proteomic studies resulted in the discovery of potential biomarkers for GH action, particularly those related with the effects of GH on aging, glucose metabolism and body composition.

**Electronic supplementary material:**

The online version of this article (doi:10.1186/s12014-017-9160-2) contains supplementary material, which is available to authorized users.

## Background

**Fig. 1:**
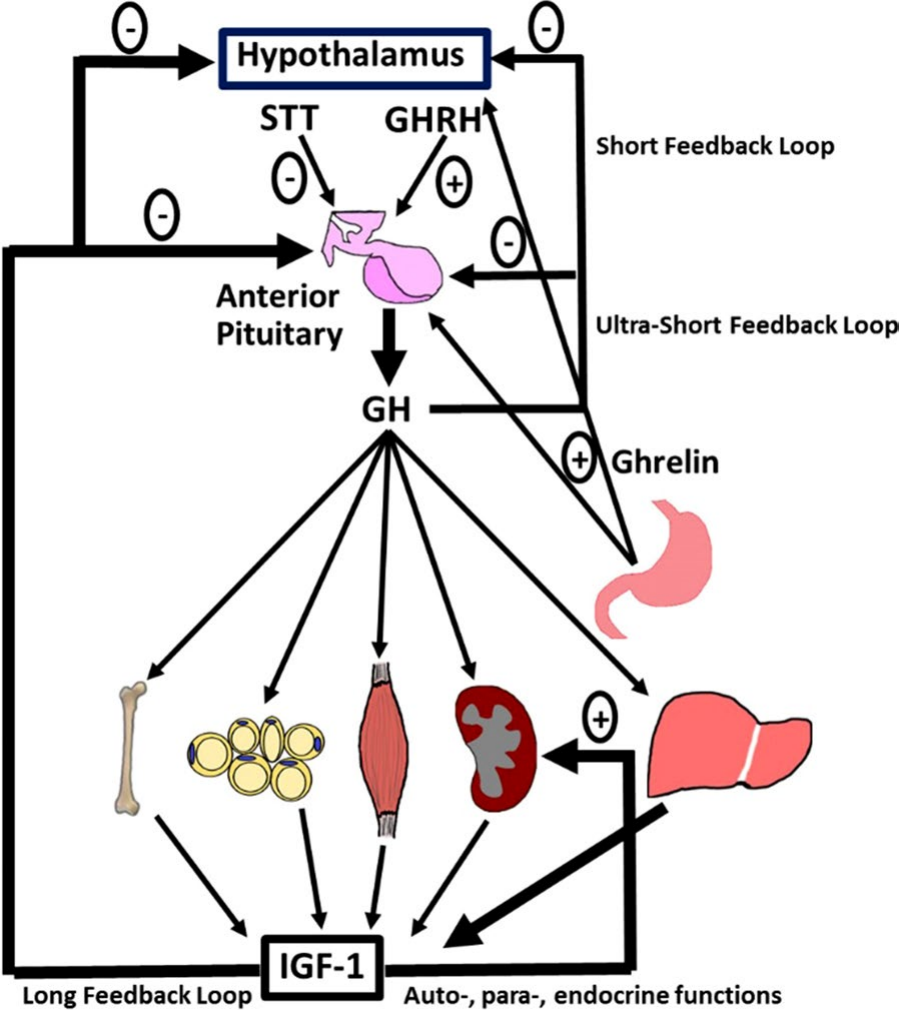
General overview of the GH/IGF-I axis. GH is secreted from the anterior pituitary in response to hypothalamic stimulus and has effects on many tissues in the body, including stimulating large amounts of IGF-I secretion by the l and other tissues. Increases in circulating IGF-I negatively impact GH release from the pituitary gland

Growth hormone (GH) is a peptide hormone secreted by somatotrophic cells of the anterior pituitary. GH has both anabolic and catabolic effects in its role as a regulator of postnatal growth and metabolism. For instance, GH promotes adipose tissue (AT) lipolysis while inducing protein synthesis in skeletal muscle, and bone growth via chondrocyte expansion in bone. GH exerts its actions by interacting with the GH receptor (GHR) and stimulating a variety of intracellular signaling pathways [1]. Cells of most tissues express GHRs on their surface; therefore GH affects most cells/tissues in the body [2]. One of the many proteins induced by GH is insulin-like growth factor-I (IGF-I), a potent growth factor that also affects many cell types. High circulating levels of IGF-I down regulate the release of GH by the anterior pituitary, a relationship that helps to define the GH/IGF-I axis (Fig. 1). Importantly, GH has both direct and indirect (via IGF-I) effects on animal growth. For example, 14% of mouse growth is a result of GH action; IGF-I promotes 35% of mouse growth; the combined action of GH and IGF-I supports 34% of mouse growth; and other factors contribute the remaining 17% to total mouse growth. Thus, GH and IGF-I have both unique and overlapping functions in terms of growth [3].

For over two decades, our laboratory has focused a portion of its research efforts on understanding GH action and its complex relationship to growth, diabetes and aging. These efforts have led us to several landmark findings including: (1) the discovery of GHR antagonists and (2) generation of the GHR gene disrupted mouse; the longest-lived laboratory mouse [4–8]. Included in these endeavors was an exploration into the proteomic fluctuations in a variety of human and mouse tissues as a function of GH action.

The aim of this review is to highlight key differences in the proteomes of several GH responsive tissues in both humans and mice. We will specifically focus on studies conducted in our laboratory using both adult humans and mouse lines with GH excess or deficiency. Prior to this undertaking, we will review techniques commonly used in the exploration of proteome composition, particularly concerning models of GH action, diabetes and aging. Through these efforts, we hope to provide a thorough and useful tool of reference for researchers working in these fields.

## Main text

### Proteomic techniques

**Fig. 2:**
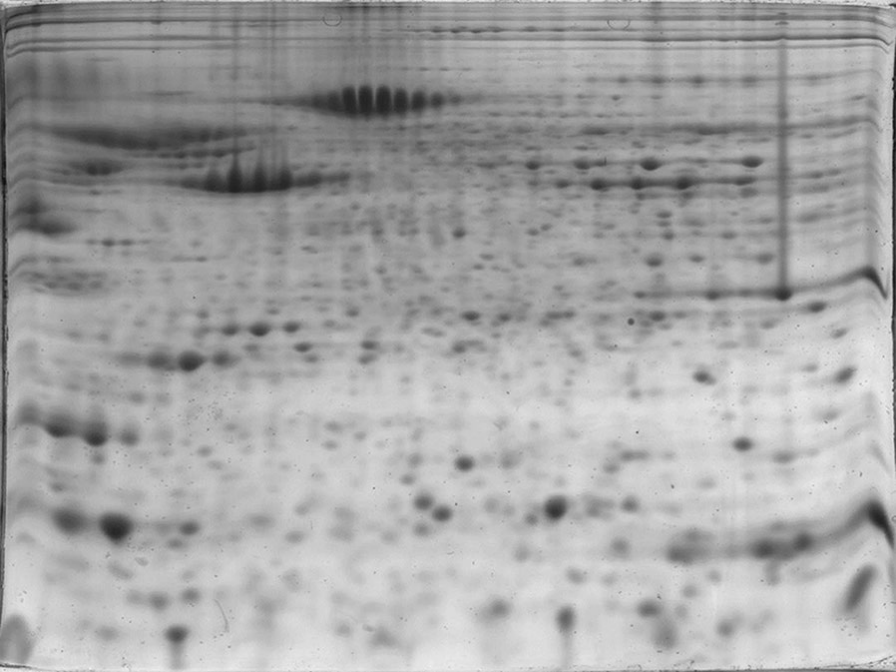
Sample 2D gel. Representative 2D gel of skin proteins in C57BL/6J mice. Image courtesy of Dr. Edward List

 The application of proteomics can be accomplished through variety of techniques including the use of antibody-based assays like the enzyme-linked immunosorbent assay (ELISA) and western blotting (WB), as well as mass spectrometry (MS) and protein arrays [9]. Another popular method is 2-dimensional gel electrophoresis (2DE), a technique that separates proteins in a sample by their isoelectric point (first dimension) and their molecular mass (second dimension). An example of a 2DE gel of proteins extracted from mouse skin is shown in Fig. 2. Although 2DE provides valuable proteomic information, it has several limitations, including difficulty with gel reproducibility, inefficiency at detecting hydrophobic proteins and proteins in low abundance, and difficulty spotting proteins with extreme molecular weights (<10 and >150 kD) or outermost pH values (pH < 3 and pH > 10) [10]. Despite these limitations, 2DE is a widely-used technique for profiling proteins and is the basis for the experiments discussed in this review.

Proteins can be separated via methods other than gel electrophoresis, including high-performance liquid chromatography (HPLC). This process relies upon pressurization by pumps, to drive a biological sample through a column, separating proteins on the basis of protein interactions with the column matrix. Protein separation by HPLC allows for a much higher resolution during the MS procedure and reduces the potential overlap between peaks of proteins occurring at the same mass. More detailed reviews of the LC–MS procedure and other types of chromatography used in proteomics can be found in the following references [11, 12].

After resolution by 2DE or chromatography, characterization and identification of proteins and their variants can be achieved by secondary techniques such as MS and/or WB. MS has been heavily used over the past two decades in the field of proteomics, with several excellent reviews written on the topic [13–15]. The MS protocol has three main components: an ion source, a mass analyzer and an ion detection system. To identify proteins using 2DE followed by MS, protein ‘spots’ are excised from the gel and digested with trypsin. The resulting fragments are then ionized, separated, and detected by the spectrometer according to their mass to charge ratio [16]. For biological samples, MS is performed primarily using one of two possible ionization sources (electrospray and matrix assisted laser desorption/ionization, or MALDI) and a number of possible mass analyzers (Quadrupole, Time of Flight, etc.) [10].

The MS experiments referenced in this review utilize MALDI as ionization source and the “time-of-flight” (TOF) as the mass analyzer. Single MALDI-TOF spectrometers determine the mass of a protein. Therefore, to determine the amino acid sequence of protein fragments, a tandem mass spectrometer (MALDI-TOF-TOF) is needed. In this tandem MS technique (MS/MS), digested peptides are mixed in a matrix solution which is then charged by a pulsed laser beam. The mixed peptides are ionized and isolated using the first TOF MS, which slows and separates peptide fragments. Individual peptide fragments are then reaccelerated and analyzed with a second TOF analyzer.

The combination of 2DE and MS/MS permits the visualization of a constellation of proteins and their post-translational modifications (PTMs). 2DE followed by MS/MS takes advantage of the high sensitivity of MS, which together with bioinformatics algorithms, allows for the unequivocal identification of a given protein. While 2DE with MS/MS has been vital for proteomic work, more recent experiments have relied upon LC–MS methods and newer MS analyzers, including Fourier transform-ion cyclotron resistance (FT-ICR) and orbitrap spectrometers. These analyzers are much quicker and higher in resolution than TOF or sector analyzers [17, 18]. Another popular proteomics technique is a multiple reaction monitoring (MRM) system referred to as MRM LC–MS, which uses a triple quadrupole MS to first isolate proteins of a specific mass to charge ratio, then fragment and analyze the selected proteins. This technique is particularly useful for serum or plasma, which contains a vast array of proteins [19]. As most of the work discussed in this review concerns results gathered using 2DE with MS/MS, further methodology sections concern only this technique.

The first step to perform 2DE is to extract the proteins from the sample that is being analyzed. Nuances in protocols for protein extraction have been described in the literature and they mainly differ depending upon the type of tissue being studied. Extracting protein from the samples is a vital step to ensure the quality of the 2DE gel results. Because the 2DE gel will essentially reflect the protein composition of the samples in terms of the molecular mass and isoelectric focusing, the main objective of protein extraction protocols is to obtain proteins from the samples without changing their physiochemical state and molecular mass. Therefore, it is important to avoid foreign protein contamination, and manipulation of the samples must be minimized in order to prevent protein degradation and modification [20].

In general, a protein extraction protocol comprises a series of steps and buffers that will solubilize, disaggregate, denature and reduce the proteins present in the sample. Hence, the protocol comprises three main steps: cell disruption, inactivation or removal of interfering substances and the solubilization of proteins [20]. Because alteration of protein charges must be avoided, charged detergents commonly used to solubilize the proteins are not used. Instead uncharged detergents and neutral molecules that disrupt water hydrogen bonds, called chaotropes, are utilized. A lysis buffer is a mixture of uncharged chemicals used to disrupt cells, and extract and solubilize the proteins from the sample [21]. A lysis buffer usually contains the neutral chaotrope urea, which disrupts the noncovalent and ionic bonds between amino acids. In some cases for more hydrophobic proteins, such as membrane proteins, urea is used in combination with thiourea [22]. To readily dissolve lipids from the samples and maintain the solubility of the proteins, electrically neutral detergents must be used [21]. One of the most common and efficient of these detergents is the zwitterionic detergent CHAPS. For nondenaturing 2DE, other nonionic detergents such as Tween 80, NP-40 and Triton X-100 are used [22]. Reducing agents are also commonly added to the lysis buffer to reduce disulfide bonds and increase solubilization of the proteins. An example of these agents are dithiothreitol (DTT) or dithioerythritol (DTE). Upon cell disruption, proteases that can degrade and affect 2DE gel results may be liberated, therefore it is advised to treat the samples with “protease inhibitor cocktails” to prevent protein degradation [22]. After the proteins have been isolated, protein concentration is determined by the Bradford assay or other methods, and the sample is prepared for loading onto gels by addition of a solubilization buffer with a reducing agent (tributylphosphine) and a sulfhydryl-alkylating agent (iodoacetamide) [23, 24].

Protocols for the extraction of protein differ upon the sample and tissue type, therefore specific combination and concentrations of chaotropes, detergents, reducing agents and proteins inhibitors need to be tested for each sample type [22, 25–27]. It is beyond the scope of this paper to discuss the specific protocols for each tissue type, and exceptional books and papers can be found for modification of general extraction protocols to adapt to specific tissue types [25–27]. In general, protein extraction of most soft tissues, heart and skin requires homogenization of the snap-frozen tissues with a biopulverizer. Lysis buffer is then added to the samples, followed by sonication and centrifugation, after which the aqueous phase containing the proteins is collected [28, 29]. There are at least two type of samples, blood and white AT (WAT), which require specific modification to extract protein properly. WAT contains high amounts of triglycerides that can make protein isolation and solubilization difficult. Therefore, for this type of tissue, thiourea is commonly used in the rehydration solution for the isoelectric focusing stage. There are several steps that can be added to the protocol to make the protein isolation from WAT more efficient. Homogenization of WAT in the lysis buffer followed by centrifugation helps to separate the lipid from the water soluble layer from the samples (where the protein is found) [30–34]. To improve cell lysis, a step of sonication in the lysis buffer can be added before centrifugation [35]. Other authors have also reported that snap freezing the WAT in liquid nitrogen followed by 48-hr long lyophilization and crushing of the tissue in a mortar using liquid nitrogen is also effective to lyse the cells [36–38]. Furthermore, the application of high and low hydrostatic pressures using pressure cycling technology has also been used to extract proteins from WAT [39]. For the studies performed in our laboratory, WAT was homogenized and sonicated in lysis buffer, followed by centrifugation and collection of the water soluble layer. We have found that delipidation by acetone or ether offers no added benefit [40, 41].

The study and identification of proteins in fluids is important as it allows the detection of biomarkers for many diseases. Protein isolation from blood starts by taking a blood sample in an anti-coagulated (i.e. heparinized) tube, followed by centrifugation 7000×*g* for 10 min to remove blood cells and collect the plasma. Body fluids such as plasma, serum, urine, or cerebral spinal fluid do not need lysing unless identification of the proteins of the blood cells is required (serum), in which case osmotic cell lysis with a hypotonic solution is sufficient [42].

A major challenge for the identification of plasma proteins is that there are a small portion of high abundance proteins that can mask the identification of less abundant ones [43]. Therefore, it is necessary to remove albumin and other high-abundance proteins from the plasma sample. Several techniques have been used to deplete albumin from the plasma including immunoaffinity resin [44]. Albumin is mainly a carrier protein, thus, one disadvantage of this method is that albumin removal also causes loss of other proteins bound to it [45]. To avoid losing proteins that can be of interest, other methods for the enrichment of less-abundant proteins can be used, such as a hexapeptide ligand library. When plasma proteins bind to their respective hexapeptide ligands, more abundant proteins will bind easily, allowing isolation of the less-abundant proteins in the sample [46]. Even though this technique avoids the loss of proteins seen in immunoaffinity, it requires about 1 ml of plasma. This large amount of sample is difficult to obtain with non-primate animals.

Another useful method to avoid the interference of albumin in 2DE gels is to perform a size exclusion by selecting for proteins with low molecular weight. Albumin’s molecular mass is ~70 kDa, and because of its high abundance, a protein smear can be seen in the 2DE gel above 50 kDa. To avoid this smear, the 2nd dimension gel with the acrylamide concentration at 15% can be prepared. In this way, proteins larger than 45 kDa remain in the upper region of the gel and the albumin smear is not found. The main advantage of this method is that it is a fast and easy way to control for the excess of albumin in the samples, but large proteins other than albumin are also removed from the analysis [47].

There are other contaminants present in plasma samples that need to be removed. For example, dialysis or precipitation can be used to remove the excess of salts that make the samples more conductive. Also, precipitation with ammonium sulfate or phenol/ammonium acetate are used to remove polysaccharides that interfere with the pores of the gel [48].

Our studies have determined that a majority of plasma proteins migrate between isoelectric points of 5–8, thus, we commonly use a 17-cm immobilized pH gradient strips (pH 3–10) for the first dimension resolution. After rehydration of the strips (50 V) for 12 h at 2 °C using a Protean IEF cell (Bio-rad), strips are used in the first dimension electrophoresis which is performed at 10,000 for 60,000 Vh. We then cut 4.5 cm from both sides of the strip to obtain an 8 cm (pH 5–8) central portion which is used for resolution in the second dimension. A current of 25 mA/gel for 250 Vh is used for this electrophoresis. This type of technique for the isoelectric focusing portion of the protocol has been found to be adequate for identification of most proteins in plasma, skin and stomach [29, 49]. For the second dimension, we perform sodium dodecyl sulfate polyacrylamide gel electrophoresis (SDS-PAGE) using 15% polyacrylamide (PA) gels. Proteins up to ~50 kDa can be resolved in this type of gel. However, a variety of PA percentages can be used ranging from 8 to 20 including gradient gels.

Fluorescent 2D difference gel electrophoresis (2D-DIGE) is a variation that overcomes some of the limitations of regular 2DE. 2D-DIGE is performed by labeling the proteins in a sample with Cyanine (Cy2, Cy3, or Cy5) dyes prior to 2DE. Because proteins are labeled individually with Cy Dyes, 3 samples can be mixed and resolved together allowing the comparison and analysis of the three samples in a single gel. 2D-DIGE increases the reproducibility of traditional 2DE by allowing samples to be resolved under the same electrophoretic conditions [50]. Other methods of protein tagging or labelling can be used, such as isobaric tag for relative and absolute quantitation (iTRAQ) or isotope-coded affinity tag (ICAT) [51, 52]. These tags are amenable for use with either 2D gels or chromatography, increasing the ability to detect proteins in low abundance.

Following electrophoresis, the gels are stained with SYPRO Orange (1:5000, Molecular Probes) for 2 h and images are captured using the laser-scanner Pharos FX Plus (Bio-Rad) with an excitation wavelength of 488 nm and an emission wavelength of 604 nm. Several software packages are available to analyze proteins resolved using 2DE gels [53–55]. The basic workflow of almost all methods consists of identifying spots by removing noise and enhancing the gel images as needed, then setting thresholds for quantification based on spot intensity. Our laboratory most commonly utilizes software called PDQuest to perform 2DE gel analysis. This software will prompt the user to import the gel images and group them into their respective treatment conditions. Substantial background can be removed and a master gel image is created that contains all the spots present in all gels. Spots are then normalized, which can be done in several ways. If normalizing by raw intensity or total spot volume, the total amount of protein in the gel is inferred by the intensity or volume of all spots on a single gel, which is compared from one gel to the next. This type of normalization requires that similar numbers of spots be present on each gel. If one gel has spots that are not found on other gels, these must be removed from the normalization procedure. 2DE gels can also be normalized by spiking samples prior to running the gel and utilizing the intensity of the spiked spots as a reflection of total protein. Once normalized, the desired spots can then be selected and quantified by intensity.

For identification of proteins after 2DE using MS, selected protein spots that are found to be altered between experimental and control tissue are excised from the PA gel using pipette tips generated by hand to correspond to the size of the spot to be excised. These tips are placed in a small amount of sterile, distilled water before the proteins are removed from the gels. Proteins are then analyzed by MS/MS and identified by comparing the results to a variety of databases such as MASCOT, Sequest, Comet, etc. [10].

Designing experiments for proteomic analysis of biological samples poses several challenges that are unique to the type of biological sample and the method of protein separation and quantification used. As mentioned previously, extraction of protein from biological samples is dependent upon each tissue that is being investigated, given that factors such as the amount of lipid present, degree of fibrosis or the existence of large amounts of a single protein (like albumin in blood samples or urea in urine samples) that can interfere with the yield and quality of the extracted protein. The type of results desired from the experiment also plays a substantial role in experimental design, as studies aiming to discern potential biomarkers of disease in humans will require larger cohorts and more stringent analysis than experiments merely aiming to establish correlations. Several experiments have been performed that address each of these nuances, and several guides have been written to improve output in clinical proteomics [56–59]. Researchers are encouraged to perform thorough literature reviews on a case-by-case basis before starting an experiment, with consideration given to the organism, cell type, impact of experimental treatments, availability of equipment for protein separation and analysis, and the nature of the results desired for each experiment.

The remaining sections of this review concern the proteomic experiments that have been conducted in our laboratory over the past two decades. Our laboratory focuses on several aspects of the GH/IGF-I pathway, including the diabetogenic nature of GH. In our transgenic mouse strains, excess GH is associated with a lean, but diabetic phenotype, while lack of GH is associated with an obese but healthy phenotype. To fully understand the nature of this interesting relationship, we will first examine the proteome of C57BL/6J mouse on a high fat diet (HFD), a model of diet induced obesity and diabetes.

### Proteomic changes in diet-induced diabetic and aging C57BL/6J mice

All transgenic mice in our laboratory are bred on the C57BL/6J background, with wild-type (WT) littermates used as controls. Studies have shown that C57BL/6J mice are predisposed to developing obesity and diabetes under high-fat diet (HFD), making them a suitable model of adult type 2 diabetes [60, 61]. To mimic a type 2 diabetic state of hyperglycemia, hyperinsulinemia and obesity, C57BL/6J mice are placed on an HFD consisting of 17% protein, 27% carbohydrate and 56% fat. Standard chow typically consists of 26% protein, 60% carbohydrates and 14% fat. When induced to diabetes on a HFD, or in states of advanced age, C57BL/6J mice exhibit increased circulating plasma insulin. There is some variation in the exact levels depending upon the method of measurement, but in general we have found that this value tends to fall between 0.5 and 1.0 ng/mL until roughly 6 months of age in normal mice on standard chow diet. At 9 months, these levels rise to ~1.5 ng/mL and may continue to rise to 2.0 ng/mL or higher for the duration of the mouse’s life. This increase is accelerated when C57BL/6J mice are placed on HFD, where insulin levels increase up to 3.0 ng/mL after 4 weeks on diet [28, 29, 62].

Changes in blood glucose are not always present in C57BL/6J mice fed standard chow. If present, hyperglycemia occurs beyond 9 months of age. On HFD, increases in blood glucose are typical after 2–4 weeks on the diet. These observations suggest that the C57BL/6J phenotype can be characterized by a state of increased diabetogenic potential, where plasma glucose levels are maintained by increased pancreatic insulin secretion. When placed on HFD or in certain cases of advanced age, the ability to regulate glucose levels is lost and the mice become diabetic [49, 63–65]. Differences in various tissue proteomes of C57BL/6J animals on HFD compared to those on standard diets are found in Table 1 and Additional file 1: Table S1. These changes help explain key differences in the phenotype of this widely used experimental mouse strain of diet induced obesity and provide a model for comparison with GH transgenic mice, which, because of the diabetogenic effect of GH, also develop diabetes.

**Table 1:** Proteomic changes in C57BL/6J mice on standard or high-fat diet

Study design	Differentially expressed proteins (gene symbols)			
Plasma proteomics of 8 male C57BL/6J mice and age matched controls on standard diet at 2, 4, 8, 12, 16 and 19 months [49]	*Increased*		*Decreased*	
	INS	TTR	ALB	SAA1
	Igκ	HP	PRDX2	
Plasma proteomics of 8 male mice placed on HFD at 3 weeks of age, analyzed at 2, 4, 6, 8, 10, 12, 16 and 20 weeks after diet induction [62]	*Increased*		*Decreased*	
	INS	KNG		
	RBP-4 (isoform-specific increases and decreases)			
	TTR (isoform-specific increases and decreases)			
	APOA1 (isoform and age specific increases and decreases)			
Cardiac proteomics of 5 male mice on HFD at 3 weeks of age analyzed after 8 weeks on diet [28]	*Increased*		*Decreased*	
	ECH1	NDPK1	TNNT2	CK
	MDH2	CRYAB	ACTC1	IDH3A
			AK1	ATP5B
	DES1 (isoform-specific increases and decreases)			
Pancreatic proteomics of 3 male mice per group started on HFD at 3 weeks, sacrificed for analysis over time at 2, 4, 8 and 16 weeks on diet [75]	*Increased*		*Decreased*	
	REG1	REG2	GSHPX1	
Skin proteomics of 4 male mice on HFD at 3 weeks of age analyzed after 16 weeks on diet [29]	*Increased*		*Decreased*	
	PDI	INS	ALDOA	
	KRT77	APOA1	GAPDH	
	PRDX6	FABP	TRSF	
	NDPK2	APOE		
	MDH1	14-3-3 Protein β		
	PGAM1	VPS29		
	PSMA1	PHB		
	LGALS7	TTR		
	NDPK1	NDPK2		
	Keratin II, type I			
	CK (isoform-specific increases and decreases) S100A10 (isoform-specific increases and decreases)			
Stomach proteomics of 4 male mice on HFD at 3 weeks of age, analyzed at 16 weeks [84]	*Increased*		*Decreased*	
	MYL3	PEBP1	ENO1	MDH1
	ATP5A1	LGALS2	PGAM1	MYL2
	LGALS7	APOA1	HSPB1	ALDH3A1
	FABP3	GKN1	CBR3	
	LDH2	PRDX2		
	ATP5B			
	TPI1 (isoform-specific increases and decreases)			

Review of proteomic changes in various tissues of C57BL/6J mice fed a high-fat diet. Certain proteins show isoform-specific regulation, where specific isoforms may be down- or up-regulated. A more detailed table containing isoform-specific regulatory patterns can be found in the additional files or in the original text

#### Plasma proteomics

Studies of the plasma proteome of C57BL/6J mice on a HFD reveal a number of changes relative to control mice that are reflective of isoform-specific regulation of target proteins in this model of diabetes [62]. For instance, total plasma transthyretin (TTR) was not significantly different between C57BL/6J mice on HF or standard chow diet. However, the amounts of several TTR isoforms were different. TTR isoform 2 and 3 became elevated over control levels as a function of age, while isoform 5 was significantly lower in HFD mice than control mice after 4 weeks. These isoforms, as well as isoforms of most other proteins mentioned in this review, are likely PTMs, as they exist at the same molecular weight but at different isoelectric points (PI’s). TTR is a protein secreted by the liver, its principal function is to carry thyroxine (T4) and retinol binding protein 4 (RBP-4) in the blood. Furthermore, TTR is part of a group of proteins called negative acute phase proteins (APPs). Serum concentration of these proteins is negatively correlated with inflammation [66, 67]. Considering the isoform-specific changes in TTR, the expression pattern of the isoforms in the context with the same overall plasma TTR should be the subject of future studies in regards to the question: does the diabetic state alter enzymatic activities that are responsible for the various PTM-TTR isoforms?

Additionally, apolipoprotein A1 (APOA1) was also elevated in the plasma of C57BL/6J mice on HFD versus standard chow at weeks 2, 4, 10 and 12. These changes were isoform-dependent as well. For example, relatively low levels of APOA1 isoforms 1 and 2, and elevated levels of APOA1 isoforms 3 and 4 were found in the HFD group at weeks 6 and 16 respectively. APOA1 is a component of the high-density lipoprotein complex (HDL) and helps to carry fats, including cholesterol, from several tissues in the body to the liver for recycling and disposal. Therefore, it is reasonable to assume that mice subject to HFD, which increases overall cholesterol levels, will increase the levels of the lipoproteins needed carry triglycerides and free fatty acid throughout the body [68]. Also, two isoforms and total plasma kininogen were increased in HFD mice.

Finally, total RBP-4 expression was also significantly upregulated in the HFD condition with four isoforms identified. RBP-4 isoform 1 decreased in the HFD group and was unchanged in the normal condition, while isoform 2, 3 and 4 increased at 3, 12 and 12 weeks on HFD respectively. RBP-4 is an adipokine, that is, a hormone secreted by the adipose tissue. Its main function is to transport retinol or vitamin A through the bloodstream. Furthermore, RBP-4 is positively associated with insulin insensitivity [69, 70].

Together, these results reveal isoform-specific regulation of proteins associated with altered lipid profiles and insulin resistance. As mentioned above, RBP-4 is associated with both insulin resistance and increased adiposity. Furthermore, RBP-4 forms a homotetramer in order to complex with TTR, extending the half-life of RBP-4 in serum [71]. Observations of both GHR^−/−^ and bGH mice confuse this association, as increased RBP-4 levels are found in the insulin sensitive yet obese GHR^−/−^ mice, and decreased concentration of RBP-4 are seen in the lean yet insulin resistant bGH mice. For this study C57BL/6J mice on HFD showed increased levels of total RBP-4, reaching significance in some isoforms as early as 4 weeks and paralleling the induction of glucose intolerance. Perhaps this is due to a relationship between RBP-4 and increased AT mass. It is also possible that there is a relationship between insulin resistance and specific RBP-4 isoforms (isoform 2 specifically). More research is needed to define these relationships.

#### Heart proteomics

A 2011 study of the heart proteome in C57BL/6J mice on HFD utilizing 2DE + MS/MS documented prominent changes in proteins related to cardiac ATP generation and structural proteins [28]. Specifically, HFD mice labeled “pre-diabetic” due to increased plasma insulin expressed decreased levels of creatine kinase (CK) isoform 1, adenylate kinase 1 and isocitrate dehydrogenase. These three proteins have been associated with reductions in mitochondrial ATP synthesis and have been implicated by other studies in the pathogenesis of diabetes [72–74]. Isoform-specific increases in the heart of HFD animals were seen in desmin, malate dehydrogenase, nucleoside diphosphate kinase, peroxisomal enoyl-coA hydratase 1 and αB-crystallin. These results suggest that a HFD/pre-diabetic state in these mice lead to changes in the cardiac proteome indicative of impaired ATP formation and structural remodeling.

#### Pancreas proteomics

Studies conducted in our laboratory in 2005 analyzed the proteome of pancreatic tissue of C57BL/6J mice on a HFD by means of 2DE + MS/MS and northern blotting [75]. Groups of 3 mice were sacrificed at 2, 4, 8 and 16 weeks of age, and 4 spots on 2DE were identified as being regulated 2-fold or more in the HFD group compared to mice fed standard chow.

These proteins included 2 isoforms of regenerating islet-derived (REG) 1 protein and REG2, which were up-regulated, and glutathione peroxidase (GSHPX1 or GPX1), which was down-regulated. REG1 and REG2 are proteins implicated in pancreatic islet regeneration and resistance to diabetes [76–79]. GSHPX1 is a selenium-based mitochondrial peroxidase found in various tissues and plays an important role as an antioxidant [80, 81]. These results suggest these diabetic HFD mice display a pancreatic proteome reflective of increased islet formation and increased oxidative stress.

#### Skin proteomics

Proteomic analysis of the skin of C57BL/6J animals has been performed in our laboratory, and revealed significant differences between animals in a diabetic state induced by HFD relative to those on a standard chow diet [29]. Isoforms of CK chain M and calpactin 1 light chain were significantly downregulated in HFD-fed C57BL/6J mice. CK, an enzyme chiefly found in muscle and brain that converts creatine to phosphocreatine, is implicated in both diabetic and obese states. Studies have shown decreased CK mRNA and activity in multiple tissues of streptozotocin-induced diabetic rats, including heart, skeletal muscle and brain [82, 83]. Other decreased proteins in the skin of diet-induced diabetic mice include aldolase A, transferrin and isoform 2 of glyceraldehyde 3-phosphate dehydrogenase (G3PDH).

Twenty-two protein spots in this ‘mouse skin’ study demonstrated significant increases in HFD mice. As expected, a large portion of these proteins were related to fat metabolism, including APOA1 and APOE precursor proteins and several isoforms of fatty acid binding protein. Malate dehydrogenase, peroxiredoxin 6 and protein disulfide-isomerase were among other significantly upregulated proteins in skin tissue of these animals. These changes in the skin proteome may be induced by diabetes, obesity, and a metabolic shift to HF diet or a combination of these items. Research into other mouse models of obesity without insulin resistance (or vice versa) will clarify the potential role of these markers as determinants of diabetes progression. If skin proteomic profiles indeed reflective various states of progression from a normal to an obese, to an insulin resistant, to a diabetic state, then perhaps this procedure could be extended to humans for charting their ‘diabetic’ status.

#### Stomach proteomics

In 2007, we analyzed the stomach proteome of C57BL/6J mice on HFD versus standard chow utilizing 2DE + MS/MS [84]. In this study, expression of 11 different proteins increased in the HFD group, and 8 proteins decreased. The greatest increases occurred in myosin light peptide 3 and ATP synthase alpha subunit isoform 1, with the largest decreases seen in enolase 1 (also called α-enolase) and cytosolic malate dehydrogenase. Other notable regulated proteins include increased APOA1 and increased fatty acid binding protein 3. Increased APOA1 and FABP3 in stomach tissue of diabetic obese mice is counterintuitive, as several reports suggest decreases in both these proteins are correlated with insulin resistance [85, 86]. Decreases in enolase 1 have been noted in cardiac tissue of aging mice, but are upregulated in several other pathologies such as Alzheimer’s disease and cancer [87, 88]. Increases in ATP5B are seen in the diabetic kidney, assumedly as a compensatory mechanism to counteract advanced glycation end-products [89]. Other regulated proteins found in this study, including decreased CBR3, indicate broad alteration of metabolic processes, a common theme found in diabetic patients [90–92].

#### Proteomics in aging

In 2011, members of our laboratory attempted to define plasma markers of aging using proteomics in a longitudinal study of C57BL/6J mice on a standard diet for 24 months [49]. Mice were bled six times over this 24-month period, recognizing that the life span of these mice is ~24–30 months. A total of 39 spots were identified by 2DE + MS/MS as being significantly regulated in an age-dependent manner, including several isoforms of albumin, TTR, immunoglobulin light chain kappa locus (Igκ), haptoglobin (HP), serum amyloid protein A-1 (SAA-1) and peroxiredoxin-2 (PRX-2). Insulin levels also increased with age.

This study is another example of isoform-dependent regulation of the mouse proteome. For instance, isoform 1 of Igκ was barely detectable from 2 to 8 months, but becomes apparent between 16 and 19 months of age. The finding of 23 albumin isoforms in this study was also very striking! The fact that certain isoforms of albumin decrease specifically during the aging of C57BL/6J mouse supports other studies that show a decrease in total albumin in aging rats and humans [93–95]. Thus, differences in albumin isoforms may be a diagnostic marker of the aging mouse.

The isoform-specific elevation of Igκ, HP and TTR shown in this study, supports other studies that document increased levels of IgA, IgG and inflammatory cytokines as well as increases in TTR during aging [93, 96–99]. Decreases in PRX-2 and SAA-1 suggest metabolic dysregulation in aging mice that could contribute to accumulation of reactive oxygen species (ROS) and amyloid plaques, leading to increased cellular apoptosis [100, 101]. Future studies on the regulation of enzymes responsible for the various protein isoforms as a function of age is warranted.

#### White adipose tissue (WAT) proteomics

Due to the close relationship between GH, lipid metabolism and obesity, our laboratory is interested in studying the characteristics of WAT both in mice and humans. WAT is not only a lipid-storage organ, but also a very plastic tissue that secretes hormones and changes its mass and cell composition depending on the environment and the stimuli received from other organs and cells in the body. It is known that not all WAT depots are equal, as AT depots respond in different manners to various stimuli. In fact, different WAT pads in the body have distinct differences in their physiology and functionality [102]. Thus, data collected from WAT samples must be analyzed in consideration of their location in the body. There are two main types of WAT depending on the localization in the body: visceral WAT, which is localized in the visceral cavity, and subcutaneous (SQ) WAT, which is localized under the skin [103]. Several studies devoted to understanding the physiological differences between visceral and SQ WAT have been performed, but few studies have addressed the molecular and functional differences between SQ WAT fat depots. To determine the proteomic differences in WAT depots, our laboratory conducted two studies, one in humans and the other in mice, using various adipose depots (Table 2; Additional file 2: Table S2) [104, 105].

**Table 2:** Proteomic changes in white adipose tissue of humans and WT mice

Study design	Differentially expressed proteins (gene abbreviations)			
*Human adipose tissue*				
Proteomics of 6 subcutaneous white adipose tissue depots (upper abdominal, lower abdominal, thigh, back, flank, and hip) of 6 female subjects [104]	*Increased*			*Decreased*
	HB (4 isoforms, back depot)	VIM (3 isoforms depot specific)	ENO1 (flank depot)	HPX (lower versus upper abdominal depot)
	ALB (3 isoforms, hip depot)	ATP5B (hip, flank and upper abdominal depots)	HSP 8 (upper abdominal depot, superficial layer)	
	HSP-β6 (hip depot, superficial layer)	HSP-60 kDa (hip depot)	SOD (flank depot)	
	FABP-4 (2 isoforms, depot and isoform specific increases and decreases)			
*White adipose tissue C57BL/6J mice*				
White adipose tissue (inguinal, retroperitoneal, mesenteric and epididymal) proteomics of 6 male mice analyzed at 12 and 24 months old [105]	*Increased at 24 months*		*Decreased at 24 months*	
	ATP5B	ENO1 (2 isoforms)	CA-III (2 isoforms)	ANXA5
	PDHE1-B	IDH3α	ALB	HBB (2 isoforms)
	E-FABP	CuZn-SOD	INS	HMW adiponectin
	ERP29	PPIA	Total adiponectin	
	APOA4	APOA1 (4 isoforms, 2 depot specific increases)		
	VIM			
	ACT			

Proteomic changes in the adipose tissue of human and mouse models, showing depot and isoform specific regulation. For human adipose tissue, the significantly regulated proteins listed are those that are different between depots, not between layers of depots. Differentially expressed proteins between those layers can be found within the original text

In a collaboration with Dr. Dexter Blome at Mount Carmel East Hospital in Columbus, Ohio, SQ AT from 6 different locations or depots in the body (upper abdominal, lower abdominal, thigh, back, flank, and hip) was taken from 6 women with a body mass index (BMI) above 25 that underwent liposuction [104]. The upper abdominal and the hip SQ WAT was further separated into 2 layers of WAT, deep and superficial. A proteomics approach using 2DE gels and MS/MS was used (Table 2). Comparisons between the 2DE gels corresponding to the 6 SQ WAT depots showed significant differences in the intensity of 21 spots. Of these spots, only 14 were identified, including hemoglobin (4 isoforms), vimentin (2 isoforms), albumin (2 isoforms), β ATP synthase, mitochondrial heat shock protein (HSP)-60 kDa, FABP-4, hemopexin, alpha-enolase and superoxide dismutase (SOD), ENO1 and HSP8. Furthermore, SQ WAT depots showed specific differences in the expression pattern of the proteins. Particularly, the back SQ WAT had increased levels of hemoglobin and decreased levels of the other 6 proteins. Flank SQ WAT showed an increased expression of alpha-enolase and SOD. Hip SQ WAT showed high levels of vimentin and HSP-60 kDa, and the lower abdominal SQ WAT had lower expression of FABP-4 and hemopexin when compared to the upper abdominal SQ WAT.

In terms of the comparison between the two layers (deep and superficial) of the upper abdominal SQ WAT, 228 spots were found in the 2DE gels, 3 of those proteins showing increased intensity in the superficial layer. These proteins corresponded to vimentin, FABP 4 and HSP 8. In the superficial SQ AT of the hips, 6 spots showed more intensity in the superficial layer. However, only 3 of the 6 spots were identified and corresponded to the proteins vimentin, HSP-β6 and albumin. The deep layer of the hip showed 5 spots that had higher intensity than in the superficial layer, with 2 of these 5 spots identified as FABP-4 and β-hemoglobin.

The second proteomics study of WAT performed in our laboratory looked at WAT of WT mice at 12 and 24 months of age (Table 2). This study examined 4 different AT depots, one subcutaneous (SQ/inguinal) and three visceral (mesenteric, epididymal and retroperitoneal) [105]. In WT tissue, protein content per gram was significantly higher in the retroperitoneal depot relative to the other depots. In general, proteins that are significantly altered in WAT of aged mice included those related to generation of ATP, glucose and lipid metabolism, lipid transport, stress resistance and cytoskeleton structure. Some of the significantly altered proteins that stood out due to their related physiological functions were alpha enolase 1 (ENO1), pyruvate dehydrogenase E1 subunit β (PDHE1-B) and ATP synthase subunit β, which were increased in the WAT of 24-month old WT mice. Higher levels of these proteins suggest an increase in ATP synthesis via glycolysis and oxidative phosphorylation. Furthermore, increases in proteins related to aerobic oxidation, such as Cu/Zn superoxide dismutase, may suggest an increase in ROS and oxidative stress in aged mice [106–108]. Older animals in all backgrounds showed increased levels of epidermal fatty acid binding protein (E-FABP) and decreased levels of the bicarbonate donor and anabolic enzyme carbonic anhydrase 3 (CA-III), suggesting increased lipolysis and decreased lipogenesis in aged mice [109, 110].

The differences in the expression profile between WAT depots is usually evaluated in terms of the comparison between visceral WAT (the WAT surrounding the internal organs) and SQ AT, with increased visceral adiposity being related with comorbidities of obesity, such as type 2 diabetes and cardiovascular disease. On the contrary, SQ AT has been considered the “good” AT depot because it is able to expand and store energy in a more effective manner [111]. Importantly, our results showed that not all the SQ AT depots are equal, and the differences in their proteome may reflect the differences in behavior and storage capacities.

### Proteomes in mouse strains with GH deficiency or GH excess

As the name implies, GH is best known for regulating postnatal growth. GH affects almost all the tissues in the body, but its main effects are exerted on AT, skeletal muscle, liver, kidney and bone. Specifically, GH promotes lipolysis, protein synthesis, fluid retention, gluconeogenesis, and regulates bone formation and bone reabsorption. The GH/IGF-I axis also impacts aging and longevity in several species [112, 113]. In general, reduction of GH/IGF-I action increases life- and health-span of these organisms [8, 113].

**Fig. 3:**
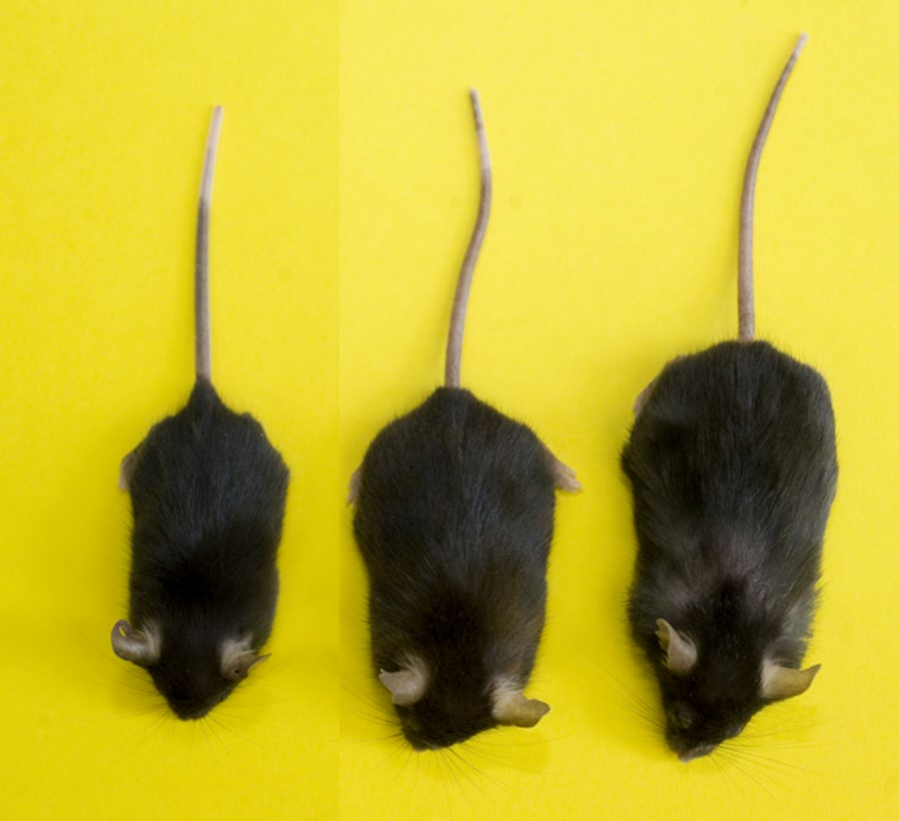
Mouse models of GH excess and deficiency. Size comparison of the GHR knockout mouse (*left*), wild-type C57BL/6J mouse (*center*) and the bGH mouse (*right*)

Two mouse models are commonly used in the study of GH action: GH transgenic mice, which overexpress GH and the GHR^−/−^ mouse, which contain loss-of function mutations in the GHR gene (Fig. 3). Our laboratory utilizes bovine (b) GH transgenic mice that express bGH cDNA under transcriptional regulation of the metallothionein promoter/enhancer. These mice are characterized by elevated circulating GH and IGF-I, which results in giant mice. bGH mice are also lean with decreased WAT [114]. However, they are insulin resistant, exhibit elevated levels of circulating insulin and develop hyperglycemia with advancing age. Furthermore, they show a 12–18 month decrease in lifespan, dying primarily from kidney, heart, and liver issues when compared to control mice (average age ~24 months) [115–118].

The GHR^−/−^ mouse was developed in our laboratory by disrupting or ‘knocking out’ the GHR gene using standard embryonic stem cell methodologies [119]. GHR^−/−^ mice are dwarf and obese. They have very low levels of IGF-I and high levels of GH, thus, they are GH resistant (or insensitive). Contrary to bGH mice, GHR^−/−^ mice have increased WAT mass, decreased lean mass, low to normal levels of glucose and low levels of insulin. Interestingly, despite this insulin sensitive phenotype, these mice appear to be less glucose tolerant when compared to controls. Even in the presence of increased WAT, GHR^−/−^ mice were found to have an increased lifespan (30–36 months) and hold the record for the longest-lived laboratory mouse [8]!

We have thoroughly discussed the phenotypes of these mice in a previous review article [8]. In parallel to human models of GH over or under expression, the phenotype of the GHR^−/−^ mouse is very similar to patients with Laron syndrome (LS), while the bGH mouse phenotype is similar to patients with gigantism and acromegaly. Patients with LS, who have inactivating mutations in the GHR gene, are obese and resistant to diabetes and cancer [63, 120–123]. Contrarily, acromegalic patients are lean, but have increased rates of diabetes and cardiovascular disease with several reports suggesting increased risk of cancer [124, 125]. Since elevated activity of the GH/IGF-I pathway has been implicated in specific types of cancer, the low levels of IGF-I found in GHR^−/−^ mice and LS patients, along with their insensitivity to GH, may be important for their relative lack of cancer [125–128].

In summary, the bGH and the GHR^−/−^ mice have opposing genotypes and phenotypes and serve as commonly utilized animal models of the human diseases acromegaly and LS. Relative to controls, the bGH mice are giant, have increased GH and IGF-I, and are lean and insulin resistant, while the GHR^−/−^ mice are dwarf, have decreased IGF-I and are obese but insulin sensitive. Several proteomic studies have been conducted in our laboratory utilizing these two mouse models. We have also performed proteomic studies on samples from patients with acromegaly, GH deficiency and patients who receive GH treatment. The results of these studies help explain phenotypic differences between models of GH excess and GH deficiency in both mouse and humans. Further elaboration on the findings of these studies can be found in the Additional file 3: Table S3, Additional file 4: Table S4, Additional file 5: Table S5, Additional file 6: Table S6, Additional file 7: Table S7, Additional file 8: Table S8.

### The bGH mouse plasma proteome

In a 2011 study, our laboratory employed 2DE followed by MS/MS to examine the plasma proteome of male bGH mice at 2, 4, 8, 12 and 16 months of age compared to WT littermates (Table 3) [64]. bGH mice exhibited significantly increased plasma levels of 5 isoforms of APOE. APOE is an apolipoprotein that carries very low density lipoproteins (VLDL), low density lipoproteins (LDL) and high density lipoproteins (HDL). APOE changes have been associated with longevity both in mice and humans, playing a role in various disease states of aging such as Alzheimer’s and atherosclerosis [129–132]. It is predictable that APOE would be increased in bGH mice, as bGH mice have higher total cholesterol levels relative to controls, and GHR^−/−^ mice have lower cholesterol levels [133, 134].

**Table 3:** Proteomic changes in the plasma of bGH mice

Study design	Differentially expressed proteins (gene abbreviations)		
*bGH mice*			
Plasma proteomics of 9 male bGH mice analyzed at 2, 4, 8, 12 and 16 months of age [64]	*Increased*		*Decreased*
	INS	IGF-I	TTR
	APOE	MBPC	
	HP	CLU	
	A2M (isoform-specific increases and decreases)		

Plasma proteome changes in bGH mice versus WT mice, showing several increased markers of inflammation

Clusterin (CLN) was also upregulated as a function of age in bGH mice and not in WT mice. CLN, also known as APOJ, has been associated with atherosclerotic plaque accumulation and renal tubular injury in both rats and humans, which could also contribute to the altered lipid profile and increased incidence of cardiovascular disease in bGH mice [135, 136]. Genome-wide association studies have found two single nucleotide polymorphisms (SNP) in the CLN gene associated with the development of Alzheimer’s disease [137, 138].

Aside from changes in APOE and APOJ (Clusterin), this study revealed that bGH mice exhibited increased expression of 4 plasma HP isoforms and one of 3 mannose-binding lectin C (MBL-C) isoforms. As acute phase immune proteins, HP binds free hemoglobin that may spill into the bloodstream following red blood cell lysis, while MBL-C plays an important role in immunity as an opsonin [139, 140].

bGH mice also displayed isoform-specific regulation of the protease inhibitor alpha-2-macroglobulin (A2 M). Alpha-2-macroglobulin (A2 M) is a proteinase inhibitor that has been shown to be protective against acute pancreatitis in mouse models [141]. Six isoforms of TTR, along with total levels of TTR, were also suppressed in bGH mice. Recall that TTR is a carrier protein for both T4 and RBP-4, and that plasma TTR concentration is known to decrease in response to inflammation. While TTR is decreased in bGH mice, it increases in WT mice as a function of age, suggesting a protective role for TTR. These results indicate increased inflammatory response in bGH mice, particularly as they age.

Complimentary studies revealed that total levels of RBP-4 were also decreased in bGH mice, with one isoform (isoform 4) increasing and one isoform (isoform 2) decreasing [47]. Recall that total RBP-4 levels were increased in C57BL/6J mice on HFD. As bGH mice are known to be lean and insulin resistant, decreases of RBP-4 are somewhat surprising. On the other hand, since RBP-4 is secreted by both the liver and WAT, the decreased mass of WAT in bGH mice may account for the decreased levels of RBP-4 found in these animals, suggesting that RBP-4 is associated more with obesity than insulin resistance.

Complementing these findings, studies performed by our colleagues have shown elevated levels of interleukin (IL)-6, tumor necrosis factor α (TNFα) and resistin, with lower levels of adiponectin in the plasma of bGH mice [142]. Together, these results suggest that bGH mice display pro-inflammatory, pro-atherosclerotic proteomic characteristics alongside a profile of insulin resistance and decreased adiposity.

### Serum proteome of patients with acromegaly

Excess GH in adulthood leads to a condition called acromegaly, which is usually caused by a functional pituitary adenoma [143]. Transsphenoidal surgery is the preferred treatment for acromegaly. Unfortunately, this treatment may not be effective when patients are not optimal candidates for the surgery, such as when adenomas are large and difficult to debulk. Furthermore, when surgery does not achieve biochemical remission (normal IGF-I level and a GH level <1.0 ng/mL during an oral glucose tolerance test) [144], medical treatment is required. The usual treatment regimen consists of various forms of somatostatin analogs such as octreotide or the GHR antagonist, Pegvisomant. Approximately, 17–35% of patients respond to somatostatin analogs with normalization of IGF-I levels, while Pegvisomant normalizes IGF-I levels in 63–95% of patients. The lower efficacy of somatostatin analogs versus pegvisomant may be due to the relative composition of somatostatin receptor subtypes in the pituitary adenoma [124, 145].

In the above 3 treatment modalities, the levels of circulating GH and IGF-I are used to assess the effectiveness of the acromegaly treatment [124]. However, these parameters are not always reliable, since discrepant results are often found (i.e. increased IGF-I and normal GH levels, or vice versa) [146, 147]. For example, a condition known as ‘micromegaly’ has been described where the patients present classic acromegalic features and have high IGF-I levels, but GH levels remain normal [148]. Because of the difficulties assessing the effectiveness of the treatments for acromegaly and GH deficiency (GHD), it is important to identify biomarkers that help reflect the effectiveness of transsphenoidal surgery and/or medical treatment.

Our laboratory has sought to determine alternate biomarkers that could be used to track treatment progress in acromegalic patients using a proteomics approach (Table 4) [149]. The objective of the study was to identify serum biomarkers that indicate the state of the acromegaly condition before and after surgery. To accomplish this objective, serum samples from 3 females and 5 males with acromegaly were collected before and 3–6 months after transsphenoidal surgery. Acromegalic patients that participated in this study were between 26–71 years of age and did not receive any treatment other than surgery. This work was performed with our colleagues at the Aarhus University Hospital in Aarhus, Denmark. Serum samples obtained were used to measure GH and IGF-I levels, as well as total HP levels. Serum collected was also used to identify proteins that significantly changed before and after surgery. Analysis was performed using 2DE gel electrophoresis, followed by protein identification by MS/MS and confirmation using WB analysis.

**Table 4:** Proteomic changes in the serum of acromegalic patients

Study design	Differentially expressed proteins (gene abbreviations)		
*Acromegaly*			
Serum proteomics of 8 acromegalic patients (3 females and 5 males), before and 3–6 months after transsphenoidal surgery [149]	*Increased*	*Decreased*	
	C4B	IGF-I	TTR (2 isoforms)
		HP a2	HBB
		APOA1 (2 isoforms)	

Significantly different changes in the serum proteome of acromegalic patients, demonstrating decreased TTR (which was also decreased in bGH mouse plasma) and increased C4B, an inflammatory marker

The results of this study showed that both the serum GH and IGF-I levels decreased significantly after surgery (*p* < 0.05). Serum levels of IGF-I were normalized in five patients that received transsphenoidal surgery. Total HP was not significantly changed in the serum of the patients before and after surgery.

Proteomic analysis revealed 7 proteins that were significantly different following transsphenoidal surgery. The intensities of 2 isoforms of TTR, HP a2, hemoglobin β subunit (HBB) and 2 isoforms of APOA1 were significantly decreased after surgery. The intensity of the protein complement C4B precursor was increased after surgery. WB analysis of serum samples was used to confirm the results obtained for HP, APOA1, and TTR, which contrarily showed that the levels of these 3 proteins were not significantly changed in serum samples after surgery.

This study showed that 7 proteins could be used as potential biomarkers for the state of the acromegaly disease. Even though the approach using 2DE + MS/MS showed that isoforms of TTR, HP and APOA1 were potential biomarkers for acromegaly treatment, 1D gel electrophoresis followed by WB analysis did not show a significant change of these proteins in serum samples of the acromegaly-treated patients. One explanation for this is that WB shows the total level of the proteins and not the level of expression of specific isoforms. Even though potential biomarkers for treatment of acromegaly were found, future studies confirming these results, as well as studies that elaborate on the specific effect of GH over these proteins, have yet to be performed.

### Serum proteome after GH Doping

The use of recombinant human GH (rhGH) by athletes for performance-enhancement is prohibited by the World Anti-Doping Agency. However, the detection of this molecule in blood is very difficult due to its short half-life (15–20 min) [150, 151]. Two tests are currently used for detection of rhGH misuse and abuse, each with their own limitations [152]. Because of these difficulties, our laboratory sought to utilize proteomic studies to identify serum biomarkers corresponding to rhGH use (Table 5) [153].

**Table 5:** Proteomic changes in the serum of patients after rhGH injection

Study design	Differentially expressed proteins (gene abbreviations)			
*rhGH doping*				
Serum proteomics of 8 male subjects treated daily for 8 days with rhGH. Serum obtained at 0, 3 and 8 days of treatment [153]	*Increased*		*Decreased*	
	ATT	TTR	APOA1	ITIH4
			HBB	

Significantly different changes in proteomes of humans after rhGH injection, showing decreases in numerous proteins produced largely in the liver

Using a randomized, cross-over design, 8 male volunteers of 23.2 ± 0.6 years old underwent two periods (8 days each) of rhGH treatment (2 mg/day at 10 PM) or placebo. A resting period of one to three weeks was given between treatments. Serum samples at 0, 3 and 8 days for both treatments were collected. 2DE followed by MS/MS was used to isolate and identify significantly altered proteins between the placebo and rhGH treatment groups, similar to our previous proteomic studies. WB was used to confirm the 2DE and MS results.

Bradford assay revealed a significant decrease in protein concentrations between placebo and GH-treated serum samples at day 8. Ninety-four spots were identified using 2DE. Of these, 5 spots showed significant variation as a function of time: alpha-1 antitrypsin (AAT), TTR, APOA1, inter-alpha-trypsin inhibitor heavy chain H4 (ITIH4) and HBB. Spot intensity corresponding to the proteins AAT and TTR increased at day 8, while APOA1, ITIH4 and HBB decreased at day 8. WB of serum samples from day 0 and day 8 of rhGH treatment were used to confirm the results. 1D WB did not show any significant changes in these proteins between day 0 and day 8 post-treatment. On the other hand, 2D WB showed these proteins as multiple spots or isoforms. Thus, the 2D WB reflected the changes seen in the 2DE + MS analyses.

In conclusion, rhGH decreased the total plasma protein concentration and significantly altered the expression of 5 plasma proteins 8 days after utilization. The reduction in total protein plasma concentration is not surprising due to the documented fluid retention promoted by GH via the renin-angiotensin-aldosterone system [154]. This fluid retention increases the plasma volume with concomitant reduction in the concentration of plasma proteins. Also, because most of the plasma proteins are secreted by the liver which has high levels of GHR expression [155], it is not surprising that 4 of the significantly altered proteins are expressed by the liver (AAT, TTR, APOA1 and ITIH4) [156–159]. More studies using a larger population and both male and female cohorts must be performed in order to validate these proteins as biomarker for rhGH doping.

### The GHR^−/−^ mouse plasma proteome

In 2012, our laboratory examined the plasma proteome of male and female GHR^−/−^ mice relative to their WT controls at 8, 16 and 24 months of age (Table 6) [160]. Contrary to bGH mice, which displayed increased protein expression of APOE, GHR^−/−^ mice displayed significantly decreased levels of total APOE over time, with many isoform-specific decreases. GHR^−/−^ mice also showed isoform-specific elevations of APOA4. APOA4 is a component of HDL and is an antiatherogenic factor [161–163]. APOA4 levels were unchanged in bGH mice, thus increases of APOA4 only in GHR^−/−^ mice may help to explain the favorable cholesterol profile found in this genotype.

**Table 6:** Proteomic changes in the plasma of GHR^−/−^ mice

Study design	Differentially expressed proteins (gene abbreviations)			
*GHR* ^−/−^ *Mice*				
Plasma proteomics of 6 male and female GHR^−/−^ mice analyzed at 8, 16 and 24 months [160]	*Increased*		*Decreased*	
	APOA4	RBP-4	MBPC	APOE
			HP	ALB
			HBB	
			IL1β (females only)	MCP1 (females only)
	APOA1 (age and isoform-specific increases and decreases)			

Proteomic changes in the plasma of the GHR knockout mouse, showing changes in several lipoproteins and decreases in inflammatory markers HP and MBPC, among others

Recall from the previous section that bGH mice exhibit increased HP and MBL-C. Opposite of this trend, GHR^−/−^ mice exhibited significantly lower levels of total plasma HP levels and MBL-C isoforms 1 and 2. These changes suggest a potential state of lower inflammation in GHR ^−/−^ mice than bGH mice when compared to WT controls. In another contrast to the bGH mouse, GHR^−/−^ mice have increased total plasma levels of RBP-4. This trend fits with the theory of RBP-4 being a marker of obesity, rather than insulin resistance, as GHR^−/−^ mice are obese but insulin sensitive.

Variations in the plasma proteome of GHR^−/−^ mice are sex-dependent and largely isoform-specific. For instance, RBP-4 isoform 2, albumin isoforms 1 and 5, and HBB isoform 1 are decreased more in female GHR^−/−^ mice than male mice. Relative to male GHR^−/−^ mice, female GHR^−/−^ mice also show decreased total plasma levels of the inflammatory cytokines IL-1β and monocyte chemotactic protein 1 (MCP1). Furthermore, female GHR^−/−^ mice exhibit lower levels of insulin than male mice [160, 164]. Taken together, our results suggest that GHR^−/−^ mice display plasma proteomic trends that likely contribute to its longer lifespan, with sex differences indicating that females have fewer inflammatory markers and changes in specific protein isoforms which are beneficial to overall health.

### The GHR^−/−^ mouse WAT proteome

As was mentioned previously, GHR^−/−^ mice have improved insulin sensitivity and increased lifespan, despite increased WAT mass. This observation is counterintuitive, as obesity and insulin insensitivity are traditionally associated with increased whole body WAT mass. Furthermore, WT mice show a significant decrease in WAT mass as they age [165]. Thus, understanding the WAT proteome and physiology of the GHR^−/−^ mice could give insight into the role of WAT on the obese but healthy phenotype of the GHR^−/−^ mice.

In 2014, our laboratory performed a study to identify proteins that differed among WAT depots of aging GHR^−/−^ and WT male mice [166]. This study used 4 AT depots of male mice at 10–12 and 22–24 months of age: one SQ/inguinal and 3 visceral (mesenteric, epididymal and retroperitoneal—Table 7). Our results suggest that protein content is lower in GHR^−/−^ mice than in WT controls in all depots except the retroperitoneal depot, which showed an increased protein content in GHR^−/−^ mice at 24 months of age.

**Table 7:** Proteomic changes in the WAT of GHR^−/−^ mice

Study design	Differentially expressed proteins (gene abbreviations)			
*GHR* ^−/−^ *adipose tissue*				
White adipose tissue (inguinal, retroperitoneal, mesenteric and epididymal) proteomics of 6 GHR^−/−^ and WT male mice analyzed at 12 and 24 months old [166]	Increased		Decreased	
	EHD2 (2 isoforms)	TTR	A2M	INS
	S100A10	HMW adiponectin	GLUT4	
	ADIPOQ	ANXA5 (retroperitoneal only)		
	HBB1 (24 mo only)			
	ACT (age and isoform specific increases and decreases)			
	APOA1 (age and isoform specific increases and decreases)			

Proteomic changes in the WAT of the GHR knockout mouse from several different depots. GHR^−/−^ mouse WAT showed increases in adiponectin, a regulator of glucose and fatty acid oxidation, as well as decreases in proteins related to glucose homeostasis

In general, there were significantly altered levels of specific proteins that may help elucidate mechanisms underlying the extended longevity phenotype of GHR^−/−^ mice. For example, expression of proteins that are involved in the endocytic process such as EH-Domain Containing 2 (Edh2), S100A10, annexin A5 and actin were significantly different in GHR ^−/−^ mice when compared to controls. It has been shown that overexpression of Edh2 inhibits the internalization of endocytic vesicles from the plasma membrane, and that in rats, inhibition of GH action increases the expression glucose transporters type 4 (GLUT4) in the plasma membrane [167]. Because this study showed upregulation of two Ehd2 isoforms in GHR ^−/−^ mice, it was hypothesized that, similar WT rats, the decrease in GH action and the increased levels of the Ehd2 isoforms may help retain GLUT4 in the membrane, thus explaining the increased insulin sensitivity seen in these mice. Therefore, GLUT4 and a full-length Ehd2 western blotting was performed. Unfortunately, no significant difference in the Ehd2 protein was seen between the two genotypes and a decreased level of GLUT4 in GHR^−/−^ WAT at 12 months of age was found.

### Serum proteome of patients with GHD

Monitoring the effects of GH action in humans is of vital importance due to the diseases associated with GH excess or deficiency. GH secretion in mammals varies during life, peaking at puberty and decreasing with age [168]. GHD is a condition caused by diminished secretion of GH from the pituitary gland and, in humans, is usually treated with daily injections of rhGH. Efficacy of the GH replacement is evaluated using anthropometric measurements (improved body weight, body composition, etc.), plasma IGF-I concentrations, lipids and glycemic profiles. Confounding factors such as preexisting metabolic abnormalities, obesity, age, gender and the dose of rhGH therapy may affect the clinical parameters used to evaluate the efficacy of GH replacement in GHD patients [169–171].

In attempt to resolve difficulties in diagnostic assessment of GHD, we conducted studies to identify serum biomarkers related to rhGH replacement in adults diagnosed with GHD [172]. Blood samples of GHD patients (n = 8, 3 females and 5 males) between 38 and 64 years of age were collected before and after 3 months of GH treatment (dose 0.2–0.4 mg/d, depending on age and gender) by our colleagues at Aarhus in Denmark. Serum samples obtained from blood were evaluated using 2DE followed by MS/MS (Table 8). IGF-I, insulin and free fatty acids were measured before and after rhGH replacement alongside measures of insulin resistance. Physical examinations (weight, height, body mass index, and body composition) were also performed.

**Table 8:** Proteomic changes in the serum of GH deficient patients

Study design	Differentially expressed proteins (gene abbreviations)		
*GH deficiency*			
Serum proteomics of 8 GH deficient patients (3 females and 5 males), before and 3 months after GH replacement therapy [172]	Increased		Decreased
	IGF-I	APOA1	HP (5 isoforms)

Proteomic changes in the plasma of GH deficient patients, showing increases in IGF-I and APOA1 alongside decreases in several HP isoforms

As expected, results showed that total serum IGF-I increased from 112 ± 11 μg/L before treatment to 179 ± 20 μg/L after GH replacement (*p* = 0.01). Insulin resistance testing, as well as free fatty acid, glucose and insulin measurements, exhibited no significant change after GH replacement. Though there was no significant change in body weight and BMI, as expected, all patients showed a significant increase in lean mass and a decrease in fat mass post-GH treatment. However, it was interesting to note that 2 of the 8 patients did not display an increase in IGF-I levels upon rhGH treatment, even though they did exhibit an increase in lean mass and a diminished fat mass. This effect is evidence to suggest that IGF-I levels may not always reflect the patient’s response to GH treatment.

In terms of the serum proteome, 111 protein spots were identified in the 2DE gels, the intensity of 6 of those spots were significantly altered. Five of the protein spots were identified by MS as isoforms of HP, all of which significantly decreased in serum samples after rhGH therapy. APOA1 was increased after GH treatment. To confirm these results, WB for HP and APOA1 were performed and showed no significant difference before and after rhGH replacement for either protein. In terms of identifying markers of GH action, we suspect changes in isoforms of the proteins are the important trend to recognize, not the total amount of the protein. We also stress the significance of the finding that two patients did not display a significant increase in IGF-I, but did exhibit changes of typical of increased GH/IGF-I axis activity.

## Discussion

Numerous differences in the phenotypes of bGH, GHR^−/−^ and C57BL/6J mice may be explained in part by proteomic variances mentioned above. However, several caveats exist which must be considered before making such correlations. First, the isoforms mentioned in this review are most often not the same: isoform 1 of RBP-4 in one paper will not necessarily be the same as isoform 1 of RBP-4 in another paper, even if they exist at similar molecular weight (MW) and pI. Determining MW and pI of various isoforms has not been standardized from one paper to the next, making comparisons between different studies conducted at different times nearly impossible without resolution of proteins in a ‘master gel’. Furthermore, understanding specific PTM’s responsible for each isoform was not within the scope of the papers reviewed in this article, and could possibly be a result of increased post-translational enzymatic activity.

The use of a master gel for several of our studies on our mouse lines has allowed us to see a small number of specific isoforms that are regulated differently by GH/IGF-I activity or normal aging (Additional file 3: Table S3, Additional file 4: Table S4, Additional file 5: Table S5, Additional file 6: Table S6, Additional file 7: Table S7, Additional file 8: Table S8). Among these protein isoforms are HP isoforms 2 and 3, which are upregulated both in bGH plasma and the plasma of aged C57BL/6J mice. Similarly, HP isoform 1 was found to be upregulated in the plasma of bGH mice, and decreased in the plasma of GHR^−/−^ females. RBP-4 isoform 2 was determined to be upregulated in GHR^−/−^ mouse plasma, while being downregulated in bGH mouse plasma. Furthermore, APOA1 isoforms 1 and 3 were decreased in the WAT of 12 month GHR^−/−^ mice, whereas these two isoforms were increased in 24-month WAT of C57BL/6J mice.

Without taking isoforms into account, several conclusions can be drawn about the general proteomic profiles in models of GH excess and deficiency when compared to models of aging and diabetes (Table 9). Among these are significant apolipoprotein changes, such as increased APOE in the plasma of bGH mice, compared to decreased APOE in GHR^−/−^ plasma and the skin of C57BL/6J mice on HFD. APOA1 was increased in the serum of patients with GHD, and decreased in acromegalic serum and the serum of patients given acute rhGH injections. An intriguing relationship, APOA1 was also decreased in GHR^−/−^ WAT but increased in WAT of aged C57BL/6J mice.

**Table 9:** Proteins regulated by several models of GH excess or deficiency

	GHR^−/−^ WAT	GHR^−/−^ plasma	GH deficiency	rhGH doping serum	bGH plasma	Acromegaly serum	HFD tissues	HFD plasma	Aging plasma	Aging WAT
APOA1	↓		↑	↓		↓	↑ skin, stomach			↑
APOE		↓			↑		↑ Skin			
APOA4		↑								↑
TTR	↑			↑	↓	↓		↑	↑	↑
HP		↓	↓		↑	↓			↑	
HBB	↑			↓						↓
RBP4		↑			↓			↑		
A2M	↓				↑↓					

Shared proteins regulated by mouse or human models of GH excess or deficiency, including several apolipoproteins and inflammatory markers. Upward-pointing arrows indicate increased expression, whereas downward-pointing arrows indicate decreased expression. Arrows in both directions represent isoform-specificity, with total levels of the protein unchanged

Regulation of TTR and RBP-4 is also interesting given previous relationships between the TTR/RBP-4 complex and diabetes. TTR was increased in both GHR^−/−^ WAT and the WAT and plasma of C57BL/6J mice on HFD or in advanced age. However, TTR was decreased in bGH plasma and the serum of patients with acromegaly. RBP-4 was increased in GHR^−/−^ mouse plasma and decreased in bGH mouse plasma, but also increased in C57BL/6J mice on a HFD. As mentioned previously, this relationship could signify that TTR and RBP-4 are increased as a measure of adiposity, regardless of the “healthiness” of the adipose tissue.

Proteins important for the inflammatory response where also significantly altered in many of our studies, including decreased HP levels at all ages in GHR^−/−^ mice and increasing HP levels as a function of age in bGH mice, as well as in the serum of aging C57BL/6J mice. However, the serum of acromegalic patients contained significantly decreased HP, a confusing finding given the increased inflammatory profile in these patients. Furthermore, isoforms of two other inflammatory proteins, A2 M and TTR, are significantly altered only in bGH mice.

Understanding the actions of the GH/IGF-I axis also requires understanding of WAT proteomics, specifically in light of the “healthy but obese” profile of GHR^−/−^ animals and human LS patients [63, 120–123]. Research on the differences in protein regulation between different WAT depots in humans and mice, both in obese and non-obese states, has suggested a more complex role for WAT than initially suggested. These observations include proteomic differences reflective of increased lipolysis, increased ROS and decreased lipogenesis in the AT of aging animals, as well as protein expression differences in SQ WAT depots of humans depending upon the tissue location. The role of each of these factors in the development of a healthy or diseased phenotype has yet to be elucidated.

Advances in proteomic technologies may help clarify the relationship between proteins identified in these studies and GH levels, diabetes and obesity. In the last few years, there have been significant advances in these technologies that increase the sensitivity and depth of proteome analysis [173]. For example, MALDI-MS imaging (MALDI-MSI) allows the detection of proteins directly from tissues sections, thus, enabling the study of proteins in a space and time context [174]. Furthermore, this technique in conjunction with tissue biopsy could help to identify a pathology in its early stages in a specific tissue. This type of technology could be particularly useful for diseases such as cancer [175].

The investigation of the role of specific proteins in specific tissues could also be of great importance for other conditions like diabetes [176]. Other advances in proteomic technologies allow for research of the proteome in specific cell populations. Laser capture microdissection-MS (LCM-MS) separates particular cell types from tissue sections, expanding the knowledge of the involvement of a particular cell type and proteome signature in a disease [173, 177]. In terms of discovery based platforms, the protein microarray is another technology that allows parallel identification of a large number of proteins that have affinities for specific ligands. Some disadvantages of this technology are the limited availability of ligands, especially for proteins with PTMs, and that the researcher may need to have an idea of the proteins that are of interest in the experimental sample before analysis [178].

After possible biomarkers for diseases have been investigated and identified, it is convenient to perform a targeted based proteomic analysis, in which specific proteins are identified and where larger cohorts and samples can be tested. Besides Western blots and ELISAs, other technologies have been developed that allow accurate quantification of specific proteins. Multiplex technology is an immunoassay that allows the use of cells, tissue extracts and biological fluids to measure and identify multiple proteins present in a sample at the same time [179]. Therefore, the study of the biomarkers can be validated not only in many samples simultaneously, but also in the context of protein interactions and networks in a particular disease. For example, some of the relevant proteins identified in our studies are known for their involvement in inflammation and ROS. With the multiplexed technology, it could be possible to identify several proteins that are relevant for a specific pathway, thus leading to an identification of a phenotype (for example tissues or animals that show an overall phenotype of inflammation of resistance to ROS).

## Conclusion

In this review, we have discussed the differences in tissue proteomic profiles between 3 commonly studied mouse models of type 2 diabetes and aging: bGH, GHR^−/−^ and C57BL/6J mice on HFD. These mice are distinctly different in regards to their size, metabolic parameters and lifespan. Our results suggest that 2DE electrophoresis in conjunction with WB is a useful methodology to confirm the results found with the 2DE + MS/MS approach. Most of the proteins identified by us are subjected to post-translational modification and, because of this, regular 1D electrophoresis and WB analysis is insufficient to identify significant changes in proteins between samples. On the other hand, 2DE electrophoresis and WB analysis can correctly identify the protein and PTMs, confirm the pI and MW, and determine the relative expression change between samples. However, a limitation to 2DE is that proteins with relatively low abundance will not be detected. Furthermore, to make adequate comparisons between changes in specific isoforms of a protein, standardized procedures must be utilized for each 2DE gel analysis, a nearly impossible task when studies are conducted in different laboratories.

Our proteomic studies suggest that differences in several proteins related with lipid metabolism, glucose metabolism and inflammation may play important roles in determining the differences in life- and health-span related to changes in the GH/IGF-I axis. These proteins, including TTR, HP, HBB and various apolipoproteins could serve as potential biomarkers of diseases related to GH excess or deficiency. Isoform specificity of these potential biomarkers must be thoroughly investigated, as it is evident that GH/IGF-I activity selectively regulates specific protein isoforms. As abnormalities in the GH/IGF-I axis can produce considerable injury to the patients they affect, utilizing these results to explore biomarkers of therapy for patients with acromegaly and GHD could potentially improve treatment, resulting in measurable improvements in both lifespan and quality of life of patients.

## Additional files

**Additional file 1: Table S1.** Detailed summary of proteomic changes found in several studies of diet-induced obesity and aging in C57BL/6J mice.

**Additional file 2: Table S2.** Detailed summary of proteomic changes found in a study of different adipose tissue depots in human adults and a study of adipose tissue in C57BL/6J mice as they age.

**Additional file 3: Table S3.** Proteomic changes in the plasma of bGH mice as they age.

**Additional file 4: Table S4.** Proteomic changes in the serum of acromegalic patients after transsphenoidal surgery.

**Additional file 5: Table S5.** Proteomic changes in adult serum after short-term rhGH treatment.

**Additional file 6: Table S6.** Proteomic changes in plasma of GHR^−/−^ mice as they age.

**Additional file 7: Table S7.** Proteomic changes in the WAT of GHR^−/−^ mice as they age.

**Additional file 8: Table S8.** Proteomic changes in the serum of adult GH deficient patients after treatment.

## Abbreviations

1D: 1 dimension
2D-DIGE: fluorescent 2D difference gel electrophoresis
2DE: 2-dimensional gel electrophoresis
2DE + MS/MS: 2-dimensional gel electrophoresis plus tandem mass spectrometry
A2M: alpha-2-macroglobulin
AAT: alpha-1 antitrypsin
ACT: actin
ACTC1: α-cardiac actin
ADIPOQ: adiponectin
AK1: adenylate kinase isoenzyme 1
ALB: albumin
ALDH3A1: aldehyde dehydrogenase family 3
ALDOA: aldolase A
ANXA5: annexin A5
APO: apolipoprotein
APOA1: apolipoprotein A1
APOE: apolipoprotein E precursor
APPs: acute phase proteins
AT: adipose tissue
ATP5A1: ATP synthase alpha subunit isoform 1
ATP5B: ATP-synthetase β subunit
ATT: alpha-1 antitrypsin
bGH: bovine growth hormone
BMI: body mass index
C4B: complement C4B precursor
CA-III: carbonic anhydrase 3
CBR3: carbonyl reductase 3
CHAPS: (3-[(3-cholamidopropyl)dimethylammonio]-1-propanesulfonate)
CK: creatine kinase
CKM: creatine kinase chain M
CLN: clusterin
CRYAB: αB-crystallin
CuZn-SOD: copper-zinc superoxide dismutase
DES1: desmin
DTE: dithioerythritol
DTT: dithiothreitol
ECH1: peroxisomal enoyl-coA hydratase
EDH2: EH-domain containing 2
E-FABP: epidermal fatty acid binding protein
ELISA: enzyme-linked immunosorbent assay
ENO1: alpha enolase 1
ERP29: endoplasmic reticulum protein 29
FABP: fatty acid-binding protein
FT-ICR: Fourier transform-ion cyclotron resistance
G3PDH/GAPDH: glyceraldehyde 3-phosphate dehydrogenase
GH: growth hormone
GHD: growth hormone deficiency
GHR^−/−^: growth hormone receptor gene-disrupted
GHR: growth hormone receptor
GKN1: gastrokine 1
GLUT4: glucose transporter type 4
GSHPX1: glutathione peroxidase
HB: hemoglobin
HBB: hemoglobin β subunit
HDL: high density lipoproteins
HFD: high-fat diet
hGH: human growth hormone
HMW adiponectin: high molecular weight adiponectin
HP a2: haptoglobin alpha 2
HP: haptoglobin
HPX: hemopexin
HSP: heat shock protein
HSP-60kDa: mitochondrial heat shock protein 60 kDa
HSPB1: heat shock protein beta-1
HSP-β6: heat shock protein β6
ICAT: isotope-coded affinity tag
IDH3A: isocitrate dehydrogenase 3
IGF-I: insulin-like growth factor-1
Igκ: immunoglobulin light chain kappa locus
IL: interleukin
INS: insulin
ITIH4: inter-alpha-trypsin inhibitor heavy chain H4
iTRAQ: isobaric tag for relative and absolute quantitation which uses isobaric
KNG: kininogen
KRT77: keratin 77, type 2 keratin kb39
LC–MS/MS: multiple reaction monitoring system
LDH2: lactate dehydrogenase 2
LDL: low density lipoprotein
LGALS2/LGALS7: galectin 2/7
LS: Laron syndrome
MALDI: matrix assisted laser desorption/ionization
MBL-C: mannose-binding lectin C
MBPC: mannose-binding protein C
MCP1: monocyte chemotactic protein 1
MDH1/MDH2: malate dehydrogenase
MRM: multiple reaction monitoring
MS/MS: tandem mass spectrometry
MS: mass spectrometry
MYL2/3: myosin regulatory light chain 2/3
NDPK1/2: nucleoside diphosphate kinase 1/2
PA: polyacrylamide
PDHE1-B: pyruvate dehydrogenase E1 subunit β
PDI: protein disulfide-isomerase
PGAM1: phosphoglycerate mutase-1
PHB: prohibitin
pI: isoelectric point
PPIA: peptidylprolyl isomerase A
PRDX2/6: peroxiredoxin 2 or 6
PRX-2: peroxiredoxin-2
PSMA1: proteasome subunit alpha type I
PTM: post-translational modification
RBP-4: retinol binding protein 4
REG: regenerating islet-derived
rhGH: recombinant human growth hormone
ROS: reactive oxygen species
S100A10: calpactin 1 light chain
SAA-1: serum amyloid protein A-1
SDS-PAGE: sodium dodecyl sulfate polyacrylamide gel electrophoresis
SNP: single nucleotide polymorphism
SOD: superoxide dismutase
SQ: subcutaneous
T4: thyroxine
TNFα: tumor necrosis factor α
TNNT2: troponin T2
TOF: time-of-flight
TPI1: triosephosphate isomerase 1
TRSF: transferrin
TTR: transthyretin
VIM: vimentin
VLDL: very low density lipoprotein
VSP29: vacuolar protein sorting 29
WAT: white adipose tissue
WB: western blot
WT: wild-type
